# Implications of Governance, Natural Resources, and Security Threats on Economic Development: Evidence from Sub-Saharan Africa

**DOI:** 10.3390/ijerph18126236

**Published:** 2021-06-09

**Authors:** Husam Rjoub, Chuka Uzoma Ifediora, Jamiu Adetola Odugbesan, Benneth Chiemelie Iloka, João Xavier Rita, Rui Miguel Dantas, Mário Nuno Mata, José Moleiro Martins

**Affiliations:** 1 Department of Accounting and Finance, Faculty of Economics and Administrative Sciences, Cyprus International University, Mersin 10, 99040 Haspolat, Turkey; 2 Marketing Department, University of Nigeria, Enugu Campus (UNEC), Enugu 400241, Nigeria; chuka.Ifediora@unn.edu.ng; 3 Department of Business Administration, Faculty of Economics and Administrative Sciences, Cyprus International University, Mersin 10, 99040 Haspolat, Turkey; odugbesanadetola@gmail.com; 4 Department of Marketing, Faculty of Management Sciences, Enugu State University of Science and Technology, Enugu 400241, Nigeria; iloka.benneth@esut.edu.ng; 5 ISCAL-Instituto Superior de Contabilidade e Administração de Lisboa, Instituto Politécnico de Lisboa, Avenida Miguel Bombarda 20, 1069-035 Lisbon, Portugal; jmrita@iscal.ipl.pt (J.X.R.); rmdantas@iscal.ipl.pt (R.M.D.); mnmata@iscal.ipl.pt (M.N.M.); zdmmartins@gmail.com (J.M.M.); 6 Instituto Universitário de Lisboa (ISCTE-IUL), Business Research Unit (BRU-IUL), 2649-026 Lisbon, Portugal

**Keywords:** government effectiveness, security, natural resources, economic development, Sub-Saharan Africa

## Abstract

Sub-Saharan African countries are known to be bedeviled with some challenges hindering the economic development. Meanwhile, some of these issues have not been exhaustively investigated in the context of the region. Thus, this study aimed at investigating the implications of government effectiveness, availability of natural resources, and security threats on the regions’ economic development. Yearly data, spanning from 2007 to 2020, was converted from low frequency (yearly) to high frequency (quarterly) and utilized. Data analysis was conducted using Dynamic heterogeneous panel level estimators (PMG and CS-ARDL). Findings show that while PMG estimator confirms a long-run causal effect of governance, natural resources, and security threats on economic development, only natural resources show a short-run causal effect with economic development, while the CS-ARDL (model 2) confirms the significance of all the variables both in the long and short-run. Moreover, the ECT coefficients for both models were found to be statistically significant at less than 1% significance level, which indicates that the systems return back to equilibrium in case of a shock that causes disequilibrium, and in addition, reveals a stable long-run cointegration among the variables in the model. Finally, this study suggests that the policy makers in SSA countries should place more emphasis on improving governance, managing security challenges, and effectively utilizing rents from the natural resources, as all these have severe implications for the economic development of the region if not addressed.

## 1. Introduction

Reports show that the Sub-Saharan African (SSA) regions did not attain a majority of the Millennium Development Goals (MDGs) [1,2,3]. The factors behind their inability to attain these goals are still prevalent in this region [3,4]. This is mainly centered on their weak implementation measures that are predominantly based on the “whatever works for you” approach to government, which is creating big challenges for the government to align its policies together and deliver set objectives [5]. In any case, once the right measures are put in place, there are still numerous opportunities available for innovation and faster and deep transformation of the continent, if many of their resources are harnessed for sustainable solutions [6,7]. With its Sustainable Development Goals (SDGs), the region maintains its commitment towards ending hunger; attaining food security; improving nutrition; promoting sustainable agriculture; restoring, protecting, and promoting the use of terrestrial ecosystems in a sustainable way; managing their forests sustainably; combating desertification; reversing and halting degradation of labial lands; halting biodiversity losses; sustainably managing water and ensuring its availability; sustainable management of its other resources, and at the same time taking necessary measures to combat climate change as well as address the issues it yields [8]. However, addressing all these challenges depends to a great extent on the capacity of their governments to embrace deep transformation that would accelerate its economic development.

On the basis of economic development, it has been documented that the economies of the Sub-Saharan economy experienced relatively stable growth in the 2000s as a result of the then-buoyant international commodity prices. In any case, the global economy has slowed down since, with the impact being that some of these countries started to experience declined balance in the international scene, resulting in increased budget deficit and accumulated debts [2,9]. As it stands now, there are changes in the economic structure of African countries. Continental reports such as UNCTAD [10] point to the intra-investment that is taking place among the African nations; this is more pronounced in the service sector, especially when it comes to transport and finance [10]. Indeed, this represents a crucial change for the region, as it is necessary to consider the fact that the African economies have for long depended on the export of their primary commodities.

Another argument has been raised by Rodrik [11], who states that the developing nations, mostly from the Sub-Saharan Africa region, are going through “premature de-industrialization”. One thing that is commonly agreed about the Sub-Saharan region is that the economies have not been able to attain their potential, considering the level of natural resource deposits in this region. The quest for natural resource extraction is known to shape economic, social, and political relations in Africa in different but complex ways. The tendency of this quest to generate a spectrum of violent conflicts is one of the most visible impacts; this includes the low-intensity tensions that occur daily in the Zambian copper belt to the large-scale insurgencies that are taking place in Nigeria’s Niger Delta region. However, natural resources are not the only cause of conflict in this region, as some of them are religious in nature (for instance, the issues caused by Boko Haram in Nigeria). On the side of conflicts emerging as a result of natural resources, they tend to emerge as a result of long-term disruption of local livelihoods, emanating both as a result of the environmental outcomes of continued extraction of resources [12,13] and also (a) illicit flow of capitals [14] and (b) tensions brought about by inequitable revenue distribution due to the absence of local participants. With the deepened complexities that come with extraction and distribution of resources, there is a natural increase on the need for intervention on how the process is governed, together with measures geared towards accomplishing these needs [15]. Therefore, the governance of these natural resources influences security and economic development in these economies.

The need to ensure sustainable and credible governance processes for natural resources was born out of the specific circumstances taking place in the local stage and broader agenda for governance across the globe. This is focused predominantly on regulating not only the extractive practices of private corporations (of which the Voluntary Principles on Security and Human Rights is a good example) but also the measures put in place by the government to manage the acquisition of these resources (for instance, Extractive Industries Transparency Initiative, EITI). Acosta [16] has described these different opinions about the governance of natural resources as the set of strategies that are designed to enhance the level of accountability and transparency for private corporations and government when licensing, exploring, extracting, contracting, generating revenues, and allocating natural resources [17,18]. The government process is further complicated by these violent conflicts, making it necessary to develop innovative strategies that can create a nexus between resources and peaceful development [19]. Though some studies have investigated the implication of natural resources as a curse or blessing, studies that investigate the security challenges bedeviling the SSA countries, and the questions regarding income from natural resources and governance in a single model is scant in the literature; hence, the motivation of this study is to fill the gap, so as to contribute to the frontiers of knowledge by investigating the implication of governance, security threats, and natural resource rent on the economic development of the SSA countries. The findings will be beneficial to the policymakers in SSA countries in guiding them to formulate policies that will address the challenges of security threats, governance, and the mismanagement of the natural resource rent.

Given the need to understand this relationship, this paper explores the implications of effective governance, natural resource, security challenges on economic development in Sub-Saharan Africa. In this exploration, the authors aim to understand how the governance of Africa’s income from natural resources and the security challenges affect the economic development. The reason for focusing on the Sub-Saharan regions of Africa is that while they are known to house some of the world’s largest deposits of natural resources, the same region is bridled with advanced levels of conflicts and poor economic development, and this aspect of SSA has not been exhaustively empirically studied. Therefore, understanding this nexus is integral for developing the right strategies and policies to improve the governance, fix security issues, and enhance economic development in the said region.

The remainder of the paper is structured as follows: the next section (Section 2) presents review of extant literature to understand the theories that guide the relationship between governance, natural resources, security challenges, and economic development. Section 3 presents the methodology employed in this study where the data description, data sources, and methods employed are discussed. The empirical findings from estimations are presented in Section 4, while the paper discussion and conclusion rounds up the paper in Section 5.

## 2. Literature Review

### 2.1. Complexities of Being Endowed with Natural Resources in Africa

A number of works have looked at the nexus between natural resources and certain outcomes (such as civil wars, economic development, and governance) across the globe. In a review of literature, Ross [20] focused on the relationship between natural resources and civil wars, pinpointing four main views. The first point was that resources yield different levels of impact on conflict. To this end, it is believed that while oil has the likelihood of increasing conflict, other agricultural products yielded almost no impact. On the second point, it was held that while “lootable” resources like diamonds did not necessarily beget conflicts, violence associated with these resources seem intractable when it does erupt. On the third note, the author stated that there are some commodities, with legal agricultural products as good examples that do not have a relationship with civil war. On the final note, there is a weak correlation between natural resources in general and the inception of civil wars.

The studies of Le Billon [21] and Snyder [22] together with other similar ones, for instance, have mainly focused on civil wars as a measure of violence. However, there are numerous everyday violent conflicts, although normally on the lower level, that is critical to consider in relation to the overall stability of states even when they do not seem to hit the international news headlines. The farmer–herdsmen conflict in Nigeria that was previously minimal [23] is a good example as its escalation has caused numerous issues in present-day Nigeria. Therefore, these examples seem to query the general assumption in literature that agricultural resources hold almost no impact on civil wars [24,25].

Alden and Alves [26], in a study that was published by the Oxford University’s Centre for the Study of African Economies (CSAE), Arezki, used a geocode data set to present the argument that, contrary to the general assumption, there is no empirical correlation between the emergence of violence and discovery of resources. However, it is actually difficult to sustain this line of argument especially as there is widespread evidence of intractable violence that apparently seems to be associated with the politics of extraction as well as social inequalities that come with them. The Democratic Republic of Congo (formerly Zaire) stands clear as a good example, with over five decades of violence that erupted because of huge mineral deposits and resulting in more three million people dead. This conflict has not only affected Congo’s development, as there are records showing that it has also created some of the most alarming environmental devastations that Africa has witnessed in the 21st century [27], featuring the depleted aquatic lives and water in the country’s river basin, which is the second largest in the world after the Amazon. Similar things can be said of the relationship between natural resource exploration and conflict in the volatile oil-rich region of the Niger Delta in Nigeria [27,28] and all other parts of the country featuring increased deaths as a result of the farmer–herder clash, and the Darfur region of Sudan [29].

To further highlight this nexus, Bradshaw [29] demonstrated that natural resources do not even need to be extracted physically before they can bring about brutal conflict. In the author’s argument, natural resources by simply being discovered have the potential to create violent contestation and this can actually prevent their extraction. This was demonstrated with the case of Darfur conflict where violence came to light even before the natural resources became a pivotal revenue earner for the country. The Mano River Union countries of Sierra Leone, Liberia, and Guinea house these kind of conflicts that are linked to exploitation of natural resources with brutal wars that occurred in the 1990s and early 2000s linked to their struggle over timber, rubber deposits, and diamonds [30,31]. Thus, existing evidence points to the fact that natural resources can actually beget national security issues and undermine economic development, and these outcomes are a result of governance.

### 2.2. Security Issues and Economic Development

For every security issue faced, the solution is bound to run into the question of cost, with the potential impact being allocation of money that should have been used for development to addressing the issues or drafting new budgets to repair the infrastructures damaged as a result of the security issues. In the Sub-Saharan context, the impact is more complex because the region is marked by poverty, social diversity, demographic pressures, and expansion of the geographic space. As a result of this, the governments face immense challenges when it comes to creating and sustaining political order. Insight on the factors behind this tragic security situation in Sub-Saharan Africa is curtailed by the little information available about this region. With the region now generating global attention, experts in the tourism sector are now attempting to track and disband the network of perpetrators behind these acts. However, their analysis provides limited information as to why these economies—among the materially poorest and least developed in the world, and until not so long ago was marked by their security and social peace—are now on the edge of collapse [32]. On the same note, although Muslims represent the majority of the population of this region, the dominant ideologies of this religion feature their categorical refusal of social, religious, or political violence. However, there have been increased religious tensions in recent years (for instance, Boko Haram in Nigeria, Cameroon, and Chat), and experts have found it difficult to offer satisfactory explanations for the present issues being faced in the Sahel. These issues have cost governments of the region huge costs, especially in the area of violence bleeding damages to their existing infrastructures [32].

While there seems to be little that can be done to avert these contemporary security issues in the region, it is still important to make efforts towards mitigating its costs. Aside from the immediate and tragic loss of lives caused by these ongoing conflicts, it is also important to accord a substantial amount of attention to the economic and political ramifications. The impact of such on democratic governance throughout the region cannot be over-emphasized. This is because addressing the challenges of underdevelopment and insecurity in the Sub-Saharan region has been accompanied with the trends of autocratic and authoritarian rules in the Niger [33] with this feature becoming increasingly entrenched in Chad [33]. The impact of increased policing and security on the economic systems cannot be ignored as well, as it would amount to increased costs and potentially undermine the region’s promises on development, serving as a motivation for actors to engage in the political economy of conflict.

Aside from the direct cost that comes with added security personnel and their activities, one also needs to look at the indirect cost of security as potentially yielding devastating effect on the limited economy of this region. The tourism industry of the Sub-Saharan region that was once bubbling is now struggling as a result of political instability, facing a near collapse as a result of the ongoing security challenges. The challenges faced by the governments of this region are huge—more significant than what they have faced in past decades [33]. Although the government seem to show capacity with respect to confronting these challenges, it is also important to highlight the obvious risk of taking these issues for granted. Even the major international interventions that are focused on security have failed when it comes to addressing deteriorating security challenges in this region, and the impacts are still evident.

In essence, there exists a negative nexus between security challenges and development in the Sub-Saharan region [33]. The security issues of the region are drawing back development through infrastructural damages and added costs of running government as well as securing these economies from further conflicts. Thus, it is important that care be taken prior to any decision to securitize or militarize the region. In a bid to further demonstrate this nexus, Ajodo-Adebanjoko [34] focused on the political economy and national security implication for resource-based conflicts in Nigeria. He found that Nigeria is submerged in conflicts over the ownership, access to, and distribution or competition of natural resources like petroleum and agricultural lands, with the resulting impact being conflicts that undermine the human rights, democracy, national security, and economy of the country. This is a case similar to those in other parts of the Sub-Saharan Africa region.

### 2.3. Governance and Economic Development: On the Side of Accountability

In the twenty-first century, accountability has become of paramount importance as it ensures effective and efficient allocation and utilization of natural resources [35,36,37]. Notwithstanding its importance, governments normally face challenges in managing these natural resources because they consider the concept of accountability as ambiguous and theory and practice seem to be disconnected [38,39]. However, there has been a global shift from top-down approaches of managing natural resources to localized measures like cooperative tactics and collaboration [40,41,42,43]. Accountability is made manifest at the core of these novel measures, and it is one of the principles that can be used to assess good governance [44,45,46]. Accountability can be defined differently. It can be viewed as: (a) the allocation and acceptance of responsibility for actions and decisions; and (b) demonstration of whether or not the accepted responsibilities have been met [47]. As a result of the need to meet the accountability target, the top-down approach has continued to manifest itself with political leaders producing reports that are in line with Global Best Practice guidelines, ignoring the influence of local communities [48]. On the same note, even when the local communities are engaged, it is not clear as to whether their views are being included in these accountability reports [49]. Therefore, although there are numerous studies on accountability as defined by civil society, states, and international organizations [50,51], little effort has been made to incorporate the localized conceptualization of this concept like participation, ownership, and trust.

On the side of natural resource management and use in Africa, accountability has been exacerbated as a result of the tension between decentralized and centralized forces, together with fragmented policies [52]. In accordance with Reed et al. [53], when nations lack the right institutional framework and fragmentation, they face challenges with ensuring accountability for their natural resources. This is one of the major problems with Sub-Saharan Africa, which is characterized by the lack of appropriate institutional frameworks and fragmentation. Even in cases where such exist, the government seems to be in control and the lack of independence of these institutions also keep them at the mercy of government’s interest. Therefore, establishing accountability in this region seems difficult. 

On ensuring effective and efficient levels of accountability across systems that are used to manage natural resources, Brewer et al. [46] pointed out that it is important to have the right process for governance. This has led to the definition of government as the systematic process of building cognitive and structural social capital based on trust and aligned with solidarity through collective cooperation and actions, creating social inclusion and cohesion of the citizenry and other stakeholders in decisions centered on fostering accountability. Graham et al. [54] offered similar view by defining the concept of governance as the interaction between the processes, structures, and traditions that determine the exercise of power and responsibilities, the process of taking decisions, and how the citizens and other stakeholders are being catered to. In any case, what is generally agreed is that a systematic understanding of how accountability births good governance is still lacking within the context of natural resource management in Africa [55]. Essentially, what is understood from these discussions is that there exists a nexus between governance and economic development, with accountability moderating this relationship [55]. That is to say, good governance based on accountability will yield a positive influence on the economic development of states.

### 2.4. The Nexus: Governance, Natural Resources, Security Threats, and Economic Development

Since the decolonization era, there have been extensive studies on the governance of natural resources and conflicts (security threats) in developing economies. African states are known to be trapped in multidimensional and multifaceted civil violence as a result of this, with Kaldor [56] and Le Billion [57] terming it as “resource war” or “oil war”, with the ongoing environmental and strategic oil conflicts in Nigeria as a classic example. The Niger Delta region in Nigeria has been experiencing this kind of war since 1994. Different scholars also point to the fact that natural resources have played pivotal roles in the history of armed conflicts, especially within the African region. Still adopting Nigeria as an example, the country’s oil wealth has been more of a problem for years, instead of a source for economic development and social security [57,58,59]. In the political agenda, themes of resource conflicts, both the security and environmental, all occupy a crucial place especially among the countries of the world endowed with natural resources [60,61]. States, communities, and grassroots are increasingly becoming conscious and this has influenced the potential and actual impacts that human activities yield on the ecosystems [62]. In the Sub-Saharan region of Africa, the complex realities of resource politics are made more manifest because of the conflict between stakeholders, especially between the international oil companies (IOCs) that are backed by the state and the communities where they operate.

In these communities where resources are deposited, supported by their civil society organizations and multinational environmental activists, governments are continuously being challenged about sustainable development and environmental protection. This is mainly as a result of these bodies becoming concerned about the damages that these explorers cause on their communities and environments [63]. This has brought about different patterns of protests, which ranged from demonstrations across the streets to taking the workers of these multinational corporations hostage. However, many of the countries in this region, which are active implementers of the traditional “custodian” paradigms, are increased, becoming authoritarian in the way they attend to the demands of these communities. Ironically, governments are known to hand over heavy state interventions on the basis of democracy, economic stability, and national security [64,65]. Considering that some of the countries in the region, such as Nigeria, are heavy producers of natural resources used in powering the global economy, post-violence impacts on economic, political, and strategic developments have brought these countries to the forefront of energy, development, security, and environmental focus both on the local and international scenes [66]. Conflicts manifest not only on these countries that are founded on the traditional custodian paradigm, but also in those that have fragile social structures imposed originally on those colonized, and inherited following independence. The intellectual elites or inheritance elites that took over governance following independence did so with enormous greed and the need to make sure that the survival of the state in a world that has increasingly become competitive were among the elements that fused together to increase the level of natural resource-based conflicts in this region [66].

On a similar note, it was asserted by Bannon and Collier [67] that countries with valuable natural resources are more likely to have violent secessionist movements, and oil is considered to be exceptionally dangerous. Good examples include Biafra (Nigeria), Aceh and West Papua (Indonesia), Katanga (Democratic Republic of Congo), and Cabinda (Angola).

Supporting this assertion, Collier, Hoeffler, and Söderbom [25] conducted research that was based on a new set of data during civil wars and conflicts that occurred from 1060 to 1999, and came up with the conclusion that main causative factor for conflict is natural resources. The explanation for this can be found in normal severe grievances such as lack of political rights, high level of inequality, and divisions in the religious and ethical dimensions of the society. Within the Sub-Saharan context and that of other developing nations, Klare [68,69] offered a good insight on extortion of natural resources, like the case of diamonds in Sierra Leone and Liberia, oil in Nigeria, timber in Cambodia, and cocaine in Colombia [25,70]. Since the end of the Civil War, resource conflicts represent one of the most explosive phenomena that have defined many African economies in their extractive sense. Since then, the majority of these economies have been forced to cope with issues like intractable conflicts [71], resource wars [68], new wars [72], states that have metamorphosed into complex political emergencies [73,74], blood diamonds, securitization of resources, and petro-violence [74]. All those negative terms are used by scholars to describe the conflicts related to resources in Africa.

Thus, it is generally agreed that the abundant natural resources in Africa, together with their management, remain the main source of conflicts in the region—including the blood diamond conflict of Sierra Leone and Liberia, Nigeria’s oil conflict in the Niger Delta region, the rebellion going on in the Northern part of Niger Republic because of uranium deposits, to the issues in the Democratic Republic of Congo and Angola, countries that are very rich in resources but seem to be constantly under conflict. The picture here seems to be that in Africa, wherever or whenever they discover extractible natural resources, conflicts will emerge between the state and host communities in relation to who should maintain ownership. Similarly, it was opined by Klare [68,69] that majority of the countries ravaged by war and under constant conflicts are rich in resources. These issues seem to arise mainly over the years to conflict extractive economies blessed with natural resources and strategic minerals; however, there is a failure to avert the possibility of declining into incapacitating war and violence, and this phenomenon is described as the “paradox of plenty” [71].

Considering its effects, violent resource-based conflicts are known to birth underdevelopment, poverty, despair, human displacement, and diseases. This is completely the opposite in situations where natural resources are used for eradicating poverty and are employed for social and economic development. The expectation is that where resources abound, those that have these resources are at the advantage of utilizing them for their own good by reinvesting the funds generated through sales and also deploying the resources where they are needed within their sphere. However, in the Sub-Saharan region, the reverse has been the case with these resources constituting the main factor behind underdevelopment [63]. Take Nigeria for instance; the country’s oil constitutes the main cause of its underdevelopment. While Nigeria is one of the most powerful countries in the African continent, the conflict at the Niger Delta region constitutes a threat to its stability and unity. While the country has produced oil for over 50 years, this has not resulted in the expected socioeconomic development. In the Niger Delta region, as well as other parts of the country, oil exploration is known to pollute the environment with spillage in most cases left unattended to (at least on the immediate level); these oil spills normally lead to fire, causing partial or total destruction of vegetation in the affected areas, and this will inflict more poverty on the communities where these issues happen [75].

Essentially, the nexus between governance, natural resources, security threat and economic development in the Sub-Saharan region is well established. It seems that where the resources abound the most, conflicts abound the most. This conflict is normally brought about by the struggles of ownership between the host communities and state (governance); the resulting effects are negative for security and economic development of these states [63]. However, the studies are mostly theoretical than empirical, thus it becomes imperative to empirically investigate the nexus among these variables with the aim of guiding the policy makers in SSA countries in ensuring a sustainable economic development in the region through the consideration of governance, security threats, and natural resources.

## 3. Methodology

### Data

This study employed a data panel that was comprised of forty-five (45) “Sub-Saharan Africa” countries and covered the period from 2007 to 2020. The period covered and the countries included are subject to the availability of data in respect of the variables utilized in the study. Meanwhile, in order to enhance the time series efficiency, owing to the short span of the available series, the data are converted to quarterly series from the original annual series via utilization of interpolation approach. Literature suggests the conversion of data in this kind of situation, and as such the approach of some studies in converting annual data to quarterly was followed [76,77]. The fundamental equation for Chow–Lin disaggregation of *n* annual data to *4n* quarterly data is presented as follows:

(1) $$\hat{y}=X{\hat{\beta }}_{a}+VC^{\prime {\left(CVC^{\prime }\right)}^{-1}{\hat{u}}_{a}}$$

where

(2) $${\hat{\beta }}_{a}={\left[X^{\prime }C^{\prime {\left(CVC^{\prime }\right)}^{-1}}CX\right]}^{-1}\;X\prime C\prime {\left(CVC^{\prime }\right)}^{-1}y_{a}\;c=\left[\begin{array}{c} 1\;1\;1\;1\;0\;0\;0\;0\ldots .\;.\;.\;0\\ 0\;0\;0\;0\;1\;1\;1\;1\ldots .\;.\;.\;0\\ .\;\;.\;\;.\;\;.\;\;.\;\;.\;\;.\;\ldots \;\;.\;\;.\;\;.\\ 0\;.\;\;.\;\;.\;\;.\;\;.\;\;.\;\;.\;\ldots 1\;1\;1\;1 \end{array}\right]$$

In Equation (2), $\hat{y}$ denotes the (4*n* × 1) vector of disaggregated quarterly data, *y_a_* is the observed (*n* × 1) vector of annual data, *X* is a (4*n* × *k*) matrix of k predictor variables, *V* represents (4*n* × 4*n*) covariance matrix of quarterly error terms *u_i_*, ${\hat{u}}_{a}=y_{a}-X_{a}{\hat{\beta }}_{a}$ is an *(n* × 1*)* vector of residuals from annual regression of the data.

In our study, governance, natural resources, and security threats were employed to determine the economic development in SSA countries. The governance (*gov*) was measured with “government effectiveness”, which captures the “perceptions of the quality of public services, the quality of the civil service and the degree of its independence from political pressures, the quality of policy formulation and implementation, and the credibility of the government’s commitment to such policies” [78]. The index ranges from −2.5 (weak) to 2.5 (strong). The natural resources (*nat_res*) were measured using the “total natural resources rents (% of GDP)” as a proxy. As for the “security threats” (*sec_thr*), it was measured using the “security threats index” which considers this variable as a threat to a state, threats such as “bombings, attacks and battle-related deaths, rebel movements, mutinies, coups, or terrorism. The security apparatus also takes into account serious criminal factors, such as organized crime and homicides, and perceived trust of citizens in domestic security”. The index of this variable ranges from low (0) to high (10). The dependent variables which is the economic development was measured using “Gross capita product” (GDP) as a proxy. The GDP was measured using the “GDP per capita (constant 2010). While the natural resources and economic growth indicators are sourced from “World Bank Development Indicators”, the government effectiveness and security threat indexes are retrieved from “the Global Economy Indicators”. Moreover, in order to ensure that the GDP conform to the measures of other variables, it was converted to natural logarithm prior to the analysis”.

## 4. Method

Our study considered the panel ARDL/PMG to be one of the frequent used heterogeneous panel data estimators, however, this model has been criticized by some studies based on its inability to address the “cross-sectional dependency” issue [79,80]. Thus, “Cross-Sectionally Augmented Autoregressive Distributed Lag (CS-ARDL)” was employed in addition to the panel ARDL/PMG [79,81]. In reference to literature, the linear and average cross-sectional of both the dependent and independent variables were combined to capture the cross-sectional correlation in the error term [79]. These authors echoed further that both the “mean group (MG)” and “pooled mean group estimation (PMG)” are utilized in the CS-ARDL estimation. Meanwhile, note of earning was sounded that the time dimension (T) should be large enough so that the cross-country unit could be calculated for each. In addition, for the validity of the estimations to be guaranteed, the lagged cross-section average should be sufficient enough for inclusion.

Moreover, extant literature suggests that time series equations are estimated separately for each unit in the MG estimates, while the coefficients across units can be estimated as the unweighted average of the calculated coefficients [81,82]. The studies opined that there is an absence of restrictions that could be imposed by MG on the cross-sectional parameters. Thus, different intercepts and coefficients could exist, owing to the affordability of the highest degree of heterogeneity by the MG. However, the technique was criticized on the ground that it could be inefficient for a small cross-country dimension (N) [83]. Moreover, MG is prone to any unit outliers who could greatly alter the mean of the unit coefficients [83,84]. Based on this viewpoint, Pesaran et al. [85] considered panel ARDL/PMG to be an alternative. Over the years, this approach has been employed widely in several empirical studies owing to its being an intermediate process between the mean and pooling methods of estimations [1,2]. According to Cavalcanti et al. [81], Odugbesan & Rjoub, [1], and Odugbesan & Rjoub [2], a two-step procedure is involved in the estimation of PMG. First is the estimation of long-term slope coefficients jointly across units in the panel via concentrated likelihood techniques. Secondly, the intercept, short-term coefficients, the speed of adjustment, and the error variance are estimated through maximum likelihood on a unit-by-unit basis owing to the long-term slope coefficient estimation. Odugbesan and Rjoub [1] stressed that the “PMG are restricted to be homogeneous across the cross-sections”, but allow for heterogeneity. Similarly, Samargandi et al. [84] observed that some consistency in the estimation of short-term coefficients across units averaging each of the unit’s coefficients is being provided in the use of the PMG approach owing to its lagged cross-units dimensions.

Meanwhile, Cavalcanti et al. [81] and Samargandi et al. [84] opined that some conditions like the existence of a long-term relationship among the variables of interest should be fulfilled before using the PMG approach. This condition can be ascertained through the examination of the negative and significance of the “error correction term” (ECT) coefficient [1,2]. In addition, the dynamic specification should be largely augmented so that the independent variables can be considered exogenous. Moreover, the resulting residuals from the estimation must be serially uncorrelated. However, some studies suggested that the selection of the preferred estimation between MG and PMG approaches should in reference to the imposition of homogenous slopes for the estimated long-term parameters.

In reference to a study, it was suggested that “if the long-term coefficients are, in fact, not equal across countries, the MG estimates of the mean of long-term coefficients are consistent in restrictions while the PMG estimates are inconsistent” [83]. However, “if the homogeneity restrictions are valid, estimators that impose cross-country constraints dominate the heterogeneous ones in terms of efficiency” [83]. However, “when the long-run coefficients are the same for individual units, both MG and PMG estimates are consistent, which only the latter is efficient” [84]. Subsequently, our study considers the PMG approach to be the best available compromise for consistency and efficiency most times in reference to some studies [1,2,81,84]. Therefore, our study empirical model is based on the panel “ARDL and CS-ARDL model specifications”. The panel ARDL model’s error correction model is mathematically presented as follows:

(3) $$\Delta y_{\mathrm{it}}=\omega_{i}+\alpha_{i}(y_{i,t-1}-\theta_{i}^{\prime }x_{i,t-1})+\sum_{j=1}^{p-1}\emptyset_{ij}{∆}_{yi,t-j}+\sum_{j=0}^{q-1}\delta_{ij}∆x_{i,t-j}+\varepsilon_{it}$$

where in Equation (3) *y_it_* is the economic development (GDP) for country *i* at time *t*. *x_it_* is a 3 × 1 vector of explanatory variables; governance (*gov*), natural resources (*nat_res*), and security threats (*sec_thr*). *θ_i_* denote “the long-term equilibrium relationship between *x_it_* and y*_it_* while *ϕ_ij_* and *δ_ij_* capture the short-term dynamics between variables”. *α_i_* represents “the speed of adjustment of the economic development to the long-term equilibrium”. Meanwhile, terms in brackets denotes the cointegration relationship between *x_ij_* and *y_it_*.

Irrespective of the exogeneity or otherwise of the independent variables, the slope heterogeneity is accounted for together with different orders of integration in the variables in the conventional panel ARDL. Meanwhile, some studies observed that if the cross-sectional correlations in the errors are not accounted for, there could be some problems [1,86]. In view of this, Chudik et al. [87] posits that the problem could be addressed through the use of panel CS-ARDL, which is believed to be sufficient owing to its augmentation of the right-hand side variables set with “cross-sectional averages” of the independent variables, dependent variables, and series of their lagged values. These additional terms are meant to address the “cross-sectional correlations in the error term”. Thus, Equation (3) is modified and presented as follows:

(4) $$\Delta y_{it}=\mu_{i}+\alpha_{i}(y_{i,t-1}-\theta_{i}^{\prime }x_{i,t-1}+\alpha_{i}^{-1}{ɲ}_{i}{\bar{y}}_{t}+\alpha_{i}^{-1}{ʎ}_{i}^{\prime }{\bar{x}}_{t})+\sum_{j=1}^{p-1}\emptyset_{ij}{∆}_{yi,t-j}+\sum_{j=0}^{q-1}\delta_{ij}∆x_{i,t-j}+\sum_{j=0}^{p-1}v_{ik}\Delta {\bar{y}}_{t-j}+\sum_{j=0}^{q-1}{ʎ}_{ik}∆{\bar{x}}_{t-j}+\varepsilon_{it}$$

The cross-section average of *y_it_* and *x_it_* are denoted by $\bar{y}$*_t_* and $\bar{x}$*_t._* In Equation (4), the short-term and long-term behavior of the “cross-sectional correlation” is distinguished. Moreover, only the level parts of “cross-sectional averages” are included in the “long-term equilibrium” relationship in parentheses which is in line with the suggestion of Eberhardt & Presbitero [88]. The long-run coefficients associated with y_it_ and *x_it_*, that is *θ_i_*, and the rate of adjustment back to equilibrium, *α_i_*, are the main coefficients of interest. In order to ensure completeness, the short-run coefficients *ϕ_ij_*_,_ and *δ_ij_* are also reported.

## 5. Empirical Findings

### 5.1. Descriptive Statistics

The descriptive statistics of our study are depicted in Table 1. From the table, it shows that the average GDP (*gdp_con*) for the SSA countries is $2532.84; the government effectiveness (*gov*) seems to be weak with an average index of −0.74; natural resources (*nat_res*) has an average of 12.85% of the GDP; while the security threats (*sec_thrt*) seems to be high, with an average index of 6.81. Further analysis of the table reveals that the standard deviation for the variables is not extremely high, which is an indication of relative dispersion of the data close to their mean, while the normality test as revealed in the table indicates uneven spread of the data.

### 5.2. Unit Root Test

Though, the validity of the panel ARDL approach is certain irrespective of the order of integration of the variables, i.e., *I(0)* or *I(1)*, however, the variables’ predictive potential should not be lost [1,2,18,85,89,90]. Thus, our study employed both LLC and IPS root tests with the view of ensuring that no variable in our model is *I(2)* as well as ensuring the validity of our model, even though some studies have shown the lack of unit root test in the pre-estimation for ARDL approach. The results for the unit root tests are presented in Table 2. The table shows that the LLC confirms all the variables to be stable at level, with the exception of security threats (*sec_thrt*) which become stable at first difference. Similarly, IPS confirms the exception of security threats to be stable at level while becoming stable at first difference. In summary, it is evident that based on the two tests, the variables in our model are either *I(0)* or *I(1)*, and none is *I(2)*, which is an indication that the model used in this study is correct.

## 6. Cointegration Test

In order to maintain a consistent long-term equilibrium between the variables utilized in this study with the aim of preventing a false gradient in our estimation, the “Westerlund Cointegration Test” [90] was performed to estimate the joint integration. This test consists of four tests, which are the G_t_ and G_a_ (first two tests) that test the null hypothesis with no common integration, and the P_t_ and P_a_ (the second two tests) that combine in at least one unit to test the alternative hypothesis [1]. The results from these tests are presented in Table 3, which shows that economic development as the dependent variable has a long-term integrative relationship with governance effectiveness, natural resources, and security threats. This is as a result of the three of the tests that reject the zero hypothesis and suggest an existence of long-term relationship. Suffice it to say that, the “Westerlund Panel Cointegration Test” shows that the variables in our model complement each other.

### Long and Short-Run Causality Estimates

As stated earlier, owing to the sensitivity of the MG to the unit outliers and potential inefficiency when the cross-units dimension is not large enough, we considered PMG estimation and present the results derived from the ECM specification in Equation (1) in Table 4 (column 2). As presented in Table 4 (column 2), the long and short-run coefficients for governance (*gov*), natural resources (*nat_res*), and security threats (*sec_thrt*) are presented. The determination of the ARDL model lag order according to Loaya and Ranciere [91] generally involves a trade-off between enough length and “over-extension”, which is due to few time-series dimensions. It is worth noting that several studies have applied different approaches to lag selection, and a sizeable number of them have imposed a common lag structure for all dimensions [87], while Arnold et al. [83] and Cavalcanti et al. [81] suggested that the lag selection should be in accordance to information criterion. In view of this, the lag selection in this study is through BIC, which is subject to a maximum lag of 2 on each of the explanatory variables in our model and resulted in the model incorporating a single period lag on each variable as presented in column 2 of Table 4.

The upper part of Table 4 shows the long-run coefficients of the independent variables in our model which indicate that government effectiveness (1.38), natural resources (0.03), and security threats (0.22) have a positive and significant long-run causal relationship with economic development in SSA countries. This finding is an indication that a change in government effectiveness, income from natural resources, and security threats will contribute significantly to the economic development of Sub-Saharan African countries in the long-run. Moreover, the short-run results as presented in Table 4 (lower part) shows that only natural resources show a significant causal relationship with economic development in the short-run; meanwhile, the finding revealed that the coefficient is significant at 10% confidence level. Lastly, the ECT coefficient (−0.06) is statistically significant at less than 1% significance level, which indicates that the systems return back to equilibrium in case of a shock that causes disequilibrium, and in addition, reveals a stable long-run cointegration among the variables in the model.

Subsequently, the validity of the PMG estimation was subjected to a cross-sectionally independent test which was examined through “cross-section dependence (CD) test”. Specifically, according to Odugbesan & Rjoub (2019), the correlation coefficients between the time-series for each of the dimensions in the panel were utilized. CD statistic is standard normally distributed under the H_0_ of cross-section independence; thus the H_0_ is rejected when the *p*-value is less than 0.05. This is an indication that the PMG estimator failure to address the “cross-units dependence” renders the accuracy of PMG estimates liable to bias. In order to address this shortcoming, the CS-ARDL approach was employed, which involves the inclusion of additional lagged cross-sectional averages of both the dependent and independent variables in the estimation.

In reference to the studies of Chudik & Pesaran [79], Eberhardt & Presbitero [88] who suggested a lag length of 2, while Chudik & Pesaran [79] opined that the lag length should not exceed 3, thus, our studyselected 2 lags for our estimation. The results are presented in column 3 of Table 4. Under the CS-ARDL estimates, the H_0_ of cross-sectional dependence in the Pesaran CD test is not rejected, which is an indication that any “cross-sectional dependence” caused by common factors have been addressed when the regression is augmented with the lagged cross-sectional averages. Owing to this, the estimates under the CS-ARDL model were preferred in this study.

The results presented in column 3 of Table 4 shows that the estimated coefficient of ECT (−0.944) is negative and significance, which is consistent with previous studies that for a system to show the ability of returning to equilibrium in the cause of a shock, the ECT coefficient must be negative and significant [1,2,18]. The negative coefficient and significance of the ECT coefficient of our estimates is an indication that our system will return back to equilibrium in the case there is shock in the model. Moreover, the negative and significance of the ECT coefficient indicate a stable long-run cointegration among the variables in the estimation. The estimates result, as presented in upper part of Table 4 (column 3) shows *GOV*, *Nat_res*, and *Sec_thrt* to have positive and significant coefficients. The result shows that a change in government effectiveness, rents from natural resources, and security threats will significantly cause positive and significant changes in the economic development of SSA countries in the long-run at 1%, 5%, and 5% significance level respectively. Differently from the PMG estimates for the short-run, the CS-ARDL shows all the variables to be significant in the short-run. It is worthy to note that the use of the CS-ARDL model, as presented in column 3 of Table 4, suggests that the problems of cross-section averages largely reduce residual cross-section dependence, as evident in the CD statistic (−0.565) and *p*-value (0.621). This is an indication that estimates from the CS-ARDL estimation are valid and devoid of any bias.

## 7. Conclusions

This study empirically examined the implications of governance, natural resources, and security threats on economic development with evidence from Sub-Saharan African countries based on dynamic heterogeneous panel estimations. In today’s globalized world, understanding this nexus is critical for ensuring and sustaining development in the Sub-Saharan African region, and the development in view is not just from the angle of economic development, but also with respect to general improvement of the welfare of citizens and residents in this region. It has become imperative to understand the underlying fundamental factors that influence economic development in this region, as well as the role that governance, natural resources, and security threats play in developmental strategies. Thus, GDP was utilized as dependent variable, while variables like effective governance, security threats, and natural resources were adopted as the independent variables, and they were measured using yearly data but were converted to quarterly to enhance the frequency, and analyzed based on PMG and CS-ARDL estimators.

The panel group analysis revealed a long-run causal relationship between government effectiveness (1.38), natural resources (0.03), and security threats (0.22) on economic development in SSA countries, a relationship that is statistically significant and positive. These findings indicate that a change in the government effectiveness, income from natural resources, and security threats will contribute significantly to the economic development of Sub-Saharan African countries in the long-run. In the short-run, only natural resources showed significant causal relationship with economic development; meanwhile, the finding revealed that the coefficient is significant at 10% confidence level. Lastly, the ECT coefficient (−0.06) is statistically significant at less than 1% significance level, which indicates that the systems return back to equilibrium in case of a shock that causes disequilibrium, and in addition, reveals a stable long-run cointegration among the variables in the model. Unfortunately, no empirical study has been conducted in the past, within the context of this study, although many extant literatures suggest similar views with the results obtained [25,57,58,59,70]. These literatures suggest that effective governance, security threats, and natural resources yield both short-run and long-run impacts on economic development of Sub-Saharan African nations.

In line with the findings from this study, it is imperative for the government and stakeholders in the region to pursue policies that yield sustainable economic development with potential long-run results, instead of focusing on short-term results. These measures should begin with improved country-level governance policy that will reduce security threats and see to the effective management of natural resources. Secondly, the cash inflow emanating as a result of this measure should be channeled to sustaining the improved security levels, human capital development, and improving policies and programs geared towards sustaining effective management of natural resources. The need to develop human capital is based on the understanding that it will drive down the effects of security threats and poor management of natural resources, as well as creating the right stream of next generation leaders that will sustain these improved country-wide developments. On the last note, the effective governance policies that are found to yield long-run effects on sustaining economic development should be further strengthened in order to ensure that the rule of law will continually prevail. This will also help enhance transparency in government dealings, reduce (potentially eradicate) corruption, avail conductive business regulatory environment, and provide citizens with the opportunity of living in a country that is free of war/terrorism. In any case, it must be stated that sustainable economic development is only attainable in the Sub-Saharan African countries only when adequate policies grounded in research are put in place to attend to the issues that have been revealed in this study [1,2].

Meanwhile, this study’s limitation liesin the choice of only governance effectiveness to measure governance, whereas governance was measured from six dimensions [1], in which governance effectiveness is just one of the dimensions. For this reason, it would be interesting to explore the other five dimensions or develop an index for better understanding the contribution of governance to the economic development of SSA countries. In addition, subsequent studies can utilize another innovative panel data estimator to confirm the validity of estimates from this study, as a way of improving the method used in this study.

## Figures and Tables

**Table 1:** Descriptive statistic.

	*GDP_Con*	*GOV*	*Nat_Res*	*Sec_Thrt*
**Mean**	2532.84	−0.74	12.85	6.81
**Maximum**	20,532.95	1.06	59.21	10.00
**Minimum**	208.07	−1.85	0.000	1.50
**Std. Dev.**	3459.39	0.610	11.40	1.72
**JB *p*-value**	1468.84	39.23	347.30	8.85
**Obs.**	2520	2520	2520	2520

Note: *GDP_con* = GDP per capita (Constant $2010), *GOV* = government effectiveness, *Nat_res* = rent from natural resources, *Sec_thrt* = security threats.

**Table 2:** Unit Root Tests.

	Levin, Lin & Chu Test		Im, Pesaran and Shin W-Stat	
Variable	Level	1st Diff.	Level	1st Diff.
*GDP_Con*	−13.03 ***	-	−9.52 ***	-
*Gov*	−5.36 ***	-	−2.77	-
*Nat_res*	−10.24 ***	-	−3.85 ***	-
*Sec_thrt*	−1.41	−16.45 ***	1.71	−12.26 ***

Ho: Panels contains unit roots. *** indicate 1% significance level.

**Table 3:** Westerlund Cointegration Test.

Test	Panel
G_t_	−2.30 *
G_a_	−4.45 **
P_t_	−22.21
P_a_	−9.54 *

Note: *, ** denotes 10% and 5% significance level respectively.

**Table 4:** Estimated results of long and short-run effects.

	PMG	CS-ARDL
No. of Lagged	1	2
Long-run Equation		
*GOV*	1.38 *** (0.04)	0.935 ***(0.029)
*Nat_res*	0.003 ** (0.001)	0.007 **(0.0037)
*Sec_thrt*	0.22 ***(0.008)	0.765 **(0.0178)
Short-run Equation		
*ECT*	−0.06 **(0.093)	−0.944 ***(0.183)
Δ *GOV*	0.064 (0.037)	0.327 ***(0.0474)
Δ *Nat_res*	0.0082 ***(0.00069)	0.13 **(0.077)
Δ *Sec_thrt*	−0.002 (0.02)	1.329 **(0.405)
*Constant*	0.39 **(0.204)	0.124 ***(1.83)
Obs	2520	2520
Pesaran CD	13.421	−0.565
*p*-Value	0.000	0.631

Note: **, *** denotes 5%, and 1% significance level respectively. Values in parentheses are standard error.

## Data Availability

The data that support the findings of this study are available from World Bank.

## References

[1] Odugbesan J.A., Rjoub H. (2019). Relationship among HIV/AIDS prevalence, human capital, good governance, and sustainable development: Empirical evidence from Sub-Saharan Africa. Sustainability.

[2] Odugbesan J.A., Rjoub H. (2020). Evaluating HIV/Aids prevalence and sustainable development in sub-Saharan Africa: The role of health expenditure. Afr. Health Sci..

[3] Safriel U. (2017). Land Degradation Neutrality (LDN) in drylands and beyond—Where has it come from and where does it go. Silva Fenn..

[4] Cha S. (2017). The impact of the worldwide Millennium Development Goals campaign on maternal and under-five child mortality reduction: Where did the worldwide campaign work most effectively?. Glob. Health Action.

[5] Mbow C., Neely C., Philip D., Mbow P.A., de Leeuw J., Catacutan D. (2015). How can an integrated landscape approach contribute to the implementation of the Sustainable Development Goals (SDGs) and advance climate-smart objectives?. Climate-Smart Landscapes: Multifunctionality in Practice.

[6] Fuso Nerini F., Tomei J., To L.S., Bisaga I., Parikh P., Black M., Borrion A., Spataru C., CastánBroto V., Anandarajah G. (2018). Mapping synergies and trade-offs between energy and the Sustainable Development Goals. Nat. Energy.

[7] Santangeli A., Di Minin E., Toivonen T., Pogson M., Hastings A., Smith P., Moilanen A. (2016). Synergies and trade-offs between renewable energy expansion and biodiversity conservation—A cross-national multifactor analysis. GCB Bioenergy.

[8] United Nations General Assembly (2015). Transforming Our World: The 2030 Agenda for Sustainable Development. https://www.unfpa.org/resources/transforming-our-world-2030-agendasustainable-development.

[9] Odugbesan J.A., Ike G., Olowu G., Adeleye B.N. (2020). Investigating the causality between financial inclusion, financial development and sustainable development in Sub-Saharan Africa economies: The mediating role of foreign direct investment. J. Public Aff..

[10] UNCTAD (2013). Economic Development in Africa Report.

[11] Rodrik D. (2016). Premature deindustrialization. J. Econ. Growth.

[12] Percival V. (1995). Environmental Scarcity and Violent Conflict: The case of South Africa.

[13] Jagger P. (2012). Environmental income, rural livelihoods, and income inequality in western Uganda. For. Trees Livelihoods.

[14] World Bank (2016). The Niger Basin Climate Resilience Investment Plan: A Shared Initiative for the Benefit of the Population–Overview. World Bank Working Paper. http://documents.worldbank.org/curated/en/945801467988882421/Le-plan-dinvestissement-pour-la-résilience-climatique-du-bassin-du-fleuve-Niger-une-initiativecommune-au-profit-des-populations-resume.

[15] Grant J.A., NadègeCompaoré W.R., Mitchell M.I. (2015). New Approaches to the Governance of Natural Resources: Insights from Africa.

[16] Acosta A. (2010). Review of Impact and Effectiveness of Transparency and Accountability Initiatives, Paper prepared for the Transparency and Accountability Initiative Workshop 14–15 October.

[17] Rjoub H., Odugbesan J.A., Adebayo T.S., Wong W.K. (2021). Investigating the Causal Relationships among Carbon Emissions, Economic Growth, and Life Expectancy in Turkey: Evidence from Time and Frequency Domain Causality Techniques. Sustainability.

[18] Odugbesan J.A., Rjoub H. (2019). The causal relationship between economic growth and remittance in MINT countries: An ARDL bounds testing approach to cointegration. J. Acad. Res. Econ..

[19] Alao A. (2007). Natural Resources and Conflict in Africa: The Tragedy of Endowment.

[20] Ross M. (2004). What Do We Know About Natural Resources and Civil War?. J. Peace Res..

[21] Le Billon P. (2001). The Political Ecology of War: Natural Resources and Armed Conflict. Political Geogr..

[22] Snyder R. (2006). Does Lootable Wealth Breed Disorder? A Political Economy of Extraction Frame-work. Comp. Political Stud..

[23] Lind J. (2003). Adaptation, conflict and cooperation in pastoralist East Africa: A case study from South Turkana, Kenya. Confl. Secur. Dev..

[24] Collier P., Hoeffler A. (2004). Greed and Grievance in Civil War. Oxf. Econ. Pap..

[25] Collier P., Hoeffler A., Söderbom M. (2001). On the Duration of Civil War. Policy Working Paper 2861.

[26] Alden C., Alves A. (2009). China and Africa’s Natural Resources: The Challenges and Implications for Development and Governance.

[27] Ako R., Uddin N., Botchway F. (2011). Governance and Resource Management. Natural Resource Investment and Africa’s Development.

[28] Iwilade A. (2014). Networks of violence and becoming: Youth and the politics of patronage in Nigeria’s oil-rich Delta. J. Mod. Afr. Stud..

[29] Bradshaw M. (2009). The Geopolitics of Energy Security. Geogr. Compass.

[30] Richards P. (1996). Fighting for the Rain Forest: War, Youth, and Resources in Sierra Leone.

[31] Vigh H. (2006). Navigating Terrains of War: Youth and Soldiering in Guinea-Bissau (Array).

[32] Eizenga D. (2019). Long Term Trends across Security and Development in the Sahel.

[33] Elischer S. (2019). Contemporary Civil-Military Relations in the Sahel.

[34] Ajodo-Adebanjoko A. (2019). Political economy and national security implications of resource-based conflicts in Nigeria. Afr. Secur. Rev..

[35] Schillemans T. (2015). Managing public accountability: How public managers manage public accountability. Int. J. Public Adm..

[36] Yeboah-Assiamah E., Muller K., Domfeh K.A. (2016). Rising to the challenge: A framework for optimising value in collaborative natural resource governance. For. Policy Econ..

[37] Musavengane R., Simatele D.M. (2016). Communitybased natural resource management: The role of social capital in collaborative environmental of tribal resources in Kwa-Zulu Natal, South Africa. Dev. South Afr..

[38] Kyamusugulwa P.M. (2013). Local ownership in community-driven reconstruction in the Democratic Republic of Congo. Community Dev..

[39] Siakwah P. (2017). Are natural resource windfalls a blessing or a curse in democratic settings? Globalised assemblages and the problematic impacts of oil on Ghana’s development. Resour. Policy.

[40] Müller K. (2012). Social Capital and Collaborative Environmental Governance: Lessons from Western Cape, South Africa.

[41] Burgos A., Mertens F. (2017). Participatory management of community-based tourism: A network perspective. Community Dev..

[42] Le T.D.N. (2017). Planning ideas that matter: Livability, territoriality, governance, and reflective practice. Community Dev..

[43] Ebhuoma E., Simatele D. (2017). Defying the odds: Climate variability, asset adaptation and food security nexus in the Delta State of Nigeria. Int. J. Disaster Risk Reduct..

[44] Benson C. (2012). Shifting accountabilities? Understanding the connections between national and provincial fisheries in Papua New Guinea. Mar. Policy.

[45] Larsen R.K., Jiwan N., Rompas A., Jenito J., Osbeck M., Tarigan A. (2014). Towards ‘hybrid accountability’ in EU biofuels policy? Community grievances and competing water claims in the Central Kalimantan oil palm sector. Geoforum.

[46] Brewer J.F., Molton K., Alden R., Guenther C. (2017). Accountability, transformative learning, and alternate futures for New England groundfish catch shares. Mar. Policy.

[47] Lockwood M., Davidson J., Curtis A., Stratford E., Rod G. (2010). Governance principles for natural resource management. Soc. Nat. Resour..

[48] Musavengane R. (2018). Toward pro-poor local economic development in Zimbabwe: The role of pro-poor tourism. Afr. J. Hosp. Tour. Leis..

[49] Andrews N. (2013). Community expectations from Ghana’s new oil find: Conceptualizing corporate social responsibility as a grassroots-oriented process. Afr. Today.

[50] Stratford E., Davidson J. (2002). Capital assets and intercultural borderlands: Sociocultural challenges for natural resource management. J. Env. Manag..

[51] Stojanovska M., Miovska M., Jovanovska J., Stojanovski V. (2014). The process of forest management plans preparation in the Republic of Macedonia: Does it comprise governance principles of participation, transparency and accountability?. For. Policy Econ..

[52] Carley M., Christie I. (2000). Managing Sustainable Development.

[53] Reed M.G., Henderson A.E., Mendis-Millard S. (2013). Shaping local context and outcomes: The role of governing agencies in collaborative natural resource management. Hum. Dimens. Wildl. Int. J..

[54] Graham J., Amos B., Plumptre T. (2003). Governance Principles for Protected Areas in the 21st Century.

[55] Musavengane R., Tantoh H.B., Simatele D. (2019). A comparative analysis of collaborative environmental management of natural resources in Sub-Saharan Africa: A study of Cameroon and South Africa. J. Asian Afr. Stud..

[56] Kaldor M. (2012). New and Old Wars: Organized Violence in a Global Era.

[57] Le Billion P. (2012). Wars of Plunder: Conflicts, Profits and the Politics of Resources.

[58] Oyefusi A. (2008). Oil and the Probability of Rebel Participation among Youth in the Niger Delta of Nigeria. J. Peace Res..

[59] Olawale I., Alao A. (2007). Youths in the Interface of Development and Security. Confl. Secur. Dev..

[60] Yates D. (1996). The Rentier State in Africa: Oil-Rent Dependency and Neocolonialism in the Republic of Gabon.

[61] Yates D. (2012). The Scramble for African Oil: Oppression, Corruption and War for Control of Africa’s Natural Resources.

[62] Ross B., Schecher A. (2011). Grand Theft Nation Ali Bongo Goes to the White House. ABC News.

[63] Hurso A.-K.A. (2018). The Politics of Natural Resources Governance in Africa: Environment, Conflict and Security Nexus in Nigeria’s Niger Delta Region. A Thesis Submitted in Partial Fulfilment of the Requirements of London South Bank University for the Degree of Doctor of Philosophy. https://openresearch.lsbu.ac.uk/download/61bb6f5799a5bc1d59e2e2bea399d442a8bf571a6cbe4841369ea741ead841d8/618165/2018_PhD_Adam.pdf.

[64] Omeje K., Omeje K. (2008). Re-Engaging Rentier Theory and Polities. Extractive Economies and Conflicts in the Global South: Multi-Regional Perspectives on Rentier Politics.

[65] Watts M., Omeje K. (2008). Anatomy of an Oil Insurgency: Violence and Militants in the Niger Delta, Nigeria. Extractive Economies and Conflicts in the Global South: Multi-Regional Perspectives on Rentier Politics.

[66] Amundsen I. (2010). Good Governance in Nigeria: A Study in Political Economy and Donor Support.

[67] Bannon I., Collier P. (2003). Natural Resources and Violent Conflict: Options and Actions. The World Bank. http://documents1.worldbank.org/curated/en/578321468762592831/pdf/282450Natural0resources0violent0conflict.pdf.

[68] Klare M.T. (2002). Resource Wars: The New Landscape of Global Conflict.

[69] Klare M.T. (2012). The Race for What’s Left.

[70] Van der Poleg F. (2011). Natural resource: Curse or blessing?. J. Econ. Lit..

[71] Vallacher R.R., Coleman P.T., Nowak A., Bui-Wrzosinska L. (2010). Dynamical Foundations of Intractable Conflict: Introduction to the Special Issue. Peace Confl. J. Peace Psychol..

[72] Kaldor M. (2005). Old Wars, Cold Wars, New Wars, and the War on Terror. Int. Polit..

[73] Duffield M. (1994). Complex Emergencies and the Crisis of Developmentalism, Institute of Development Studies Bulletin: Linking Relief and Development. IDS Bull..

[74] Collier P., Lani E., HåvardHegre A., Reynal-Querol M., Sambanis N. (2003). Breaking the Conflict Trap: Civil War and Development Policy.

[75] UNEP (2011). Decoupling Natural Resource Use and Environmental Impacts from Economic Growth. https://wedocs.unep.org/handle/20.500.11822/9816.

[76] Abeysinghe T., Rajaguru G. (2004). Quarterly real GDP estimates for China and ASEAN4 with a forecast evaluation. J. Forecast..

[77] Chow G.C., Lin A.L. (1971). Best linear unbiased interpolation, distribution, and extrapolation of time series by related series. Rev. Econ. Stat..

[78] The Global Economy (2021). Business and Economic Data. https://www.theglobaleconomy.com/download-data.php.

[79] Chudik A., Pesaran M.H. (2015). Common correlated effects estimation oftheterogeneous dynamic panel data models with weakly exogenous regressors. J. Econ..

[80] Wooldridge J.M. (2002). Inverse probability weighted M-estimators for sample selection, attrition, and stratification. Port. Econ. J..

[81] Cavalcanti D.V., Tiago V., Mohaddes K., Raissi M. (2015). Commodity price volatility and the sources of growth. J. Appl. Econ..

[82] Pesaran M.H. (2015). Time Series and Panel Data Econometrics.

[83] Arnold J., Bassanini A., Scarpetta S. (2011). Solow or Lucas? Testing speed of convergence on a panel of OECD countries. Res. Econ..

[84] Samargandi N., Fidrmuc J., Ghosh S. (2015). Is the relationship between financial development and economic growth monotonic? Evidence from a sample of middle-income countries. World Dev..

[85] Pesaran M.H., Schuermann T., Weiner S.M. (2004). Modeling regional interdependencies using a global error-correcting macroeconometric model. J. Bus. Econ. Stat..

[86] Phillips P.C., Sul D. (2003). Dynamic panel estimation and homogeneity testing under cross section dependence. Econ. J..

[87] Chudik A., Mohaddes K., Pesaran M.H., Raissi M. (2013). Debt, inflation and growth: Robust estimation of long-run effects in dynamic panel data models. Tech. Report.

[88] Eberhardt M., Presbitero A.F. (2015). Public debt and growth: Heterogeneity and non-linearity. J. Int. Econ..

[89] Pesaran M.H., Shin Y., Smith R.P. (1999). Pooled mean group estimation of dynamic heterogeneous panels. J. Am. Stat. Assoc..

[90] Persyn D., Westerlund J. (2008). Error-correction–based cointegration tests for panel data. Stata J..

[91] Loayza N., Ranciere R. (2006). Financia. development, financial fragility, and growth. J. Moneycredit Bank..

